# Cabozantinib Inhibits Photodynamic Therapy-Induced Auto- and Paracrine MET Signaling in Heterotypic Pancreatic Microtumors

**DOI:** 10.3390/cancers12061401

**Published:** 2020-05-29

**Authors:** Mans Broekgaarden, Ahmed Alkhateeb, Shazia Bano, Anne-Laure Bulin, Girgis Obaid, Imran Rizvi, Tayyaba Hasan

**Affiliations:** Wellman Center for Photomedicine, Department of Dermatology, Harvard Medical School/Massachusetts General Hospital, 40 Blossom Street, Boston, MA 02114, USA; mans.broekgaarden@univ-grenoble-alpes.fr (M.B.); aalkhateeb85@gmail.com (A.A.); sbano@mgh.harvard.edu (S.B.); anne-laure.bulin@esrf.fr (A.-L.B.); obaid.girgis@mgh.harvard.edu (G.O.); imran.rizvi@unc.edu (I.R.)

**Keywords:** pancreatic cancer, photochemotherapy, combination therapy, scatter factor, cancer-associated fibroblasts, cancer stroma signaling

## Abstract

Extensive desmoplasia is a hallmark of pancreatic ductal adenocarcinoma (PDAC), which frequently associates with treatment resistance. Recent findings indicate that a combination of photodynamic therapy and the multi-kinase inhibitor cabozantinib achieved local tumor control and a significant decrease in tumor metastases in preclinical PDAC models, but the underlying therapeutic mechanisms remain unclear. This study elucidates the molecular basis of this multi-agent regimen, focusing on the role of MET signaling. Since MET activation stems from its interaction with hepatocyte growth factor (HGF), which is typically secreted by fibroblasts, we developed heterotypic PDAC microtumor models that recapitulate these interactions. In these models, MET signaling can be constitutively activated through paracrine and autocrine mechanisms. Photodynamic therapy caused significant elevations in HGF secretion by fibroblasts, suggesting it plays a complex role in the modulation of the paracrine HGF–MET signaling cascade in desmoplastic tumors. Blocking MET phosphorylation with adjuvant cabozantinib caused a significant improvement in photodynamic therapy efficacy, most notably by elevating spheroid necrosis at low radiant exposures. These findings highlight that adjuvant photodynamic therapy can augment chemotherapy efficacies, and potentially achieve improved management of desmoplastic PDAC in a more tolerable manner.

## 1. Introduction

Despite recent therapeutic advances, patients with pancreatic ductal adenocarcinoma (PDAC) are confronted with a dismal prognosis and a 5-year survival rate of approximately 5% [1]. The resistant nature of PDAC requires high-dose chemotherapeutic regimens, such as FOLFIRINOX, that are poorly tolerated and can only be administered to patients with sufficient performance status [2,3,4]. Alternatively, gemcitabine combined with albumin-bound paclitaxel (GEM-NAB) is a viable option for patients with metastatic PDAC [5]. A recent retrospective study compared FOLFIRINOX to GEM-NAB for the treatment of metastatic PDAC, which demonstrated that FOLFIRINOX was more effective in extending overall survival, albeit with a substantially higher toxicity profile [6]. The high toxicity noted for FOLFIRINOX is also observed for cabozantinib (XL-184), a multi-receptor tyrosine kinase inhibitor (RTKi) of MET, AXL, RET, and vascular endothelial growth factor receptor 2 (VEGFR2) [7]. Despite promising preclinical results [8,9], a recent Phase I clinical trial observed dose-limiting toxicity at low cabozantinib doses, and the trial was prematurely stopped as a consequence [10]. Adjuvant strategies that augment the efficacy of these regimens are, therefore, imperative to achieve better management of PDAC at lower chemotherapy doses.

In this context, photodynamic therapy (PDT) has achieved promising clinical results for the treatment of PDAC [11]. PDT involves the selective activation of a photosensitizing agent in tumor tissues by light irradiation, resulting in the local generation of reactive molecular species that cause severe oxidative stress, vascular damage followed by tumor anoxia, and the onset of anti-tumor immune responses [12,13,14,15]. Through these distinctive mechanisms and with non-overlapping toxicities, (neo)adjuvant PDT was shown to augment the efficacy of gemcitabine, oxaliplatin, irinotecan, metformin, and EGFR-inhibitors in preclinical cancer models [16,17,18,19,20,21,22,23]. Our group recently developed a photoactivatable drug delivery system containing the photosensitizer benzoporphyrin derivative (BPD) and cabozantinib. This nanoliposomal formulation enabled a PDT–RTKi combination that significantly reduced local and metastatic tumor burdens [24]. Importantly, the combination therapy yielded efficacious PDAC eradication with less than a thousandth of the clinical oral cabozantinib dose needed, underscoring that PDT can alleviate dose-limiting cabozantinib toxicities [24].

The improved treatment outcomes observed with the combination of BPD–PDT and cabozantinib were attributed to the concomitant inhibition of MET and VEGFR2 signaling by cabozantinib. However, the MET pathway is typically activated through paracrine cancer–stroma signaling, yet this interaction has not been investigated further to date. In fact, the importance of the MET signaling pathway in relation to cancer cell survival following PDT remains largely unexplored. Therefore, the current study aimed to obtain more detailed insights into the activation mechanism of this survival pathway by leveraging heterotypic 3D culture models to recapitulate the intracellular hepatocyte growth factor (HGF)–MET signaling axis. Our findings indicate that PDT can promote tumor–stroma crosstalk by promoting HGF release from fibroblasts. A combination of PDT with cabozantinib prevented MET phosphorylation and improved overall treatment outcomes.

## 2. Results

### 2.1. Characterization of Heterotypic Spheroids Comprising PDAC Cells and Fibroblasts

Heterotypic 3D culture models comprising PDAC cell lines and HGF-secreting fibroblasts were established to recapitulate the activation of the HGF–MET signaling axis as a result of tumor–stroma interactions. In these models, the MRC5 fibroblast cell line was used since it is known to secrete high levels of HGF [25]. We, therefore, explored the effects of MRC5 on the PDAC cell lines AsPC-1 (metastatic origin, high MET expression) and MIA PaCa-2 (primary cancer origin, low MET expression), which were co-cultured as suspended spheroids in ultra-low attachment plates.

**Figure 1:**
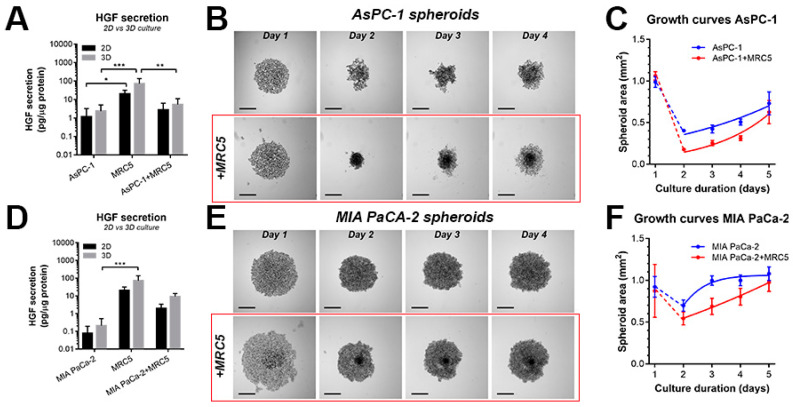
Establishment and characterization of heterotypic pancreatic ductal adenocarcinoma (PDAC) spheroids. (**A**) Quantification of hepatocyte growth factor (HGF) secretion in the conditioned media (48 h) of AsPC-1, MRC5, and AsPC-1+MRC5 2D monolayer (co-) cultures (grey bars) or 3D spheroid (co-)cultures (black bars). Depicted are the mean ± SEM of N = 6–10 from ≥3 technical repeats. (**B**) Brightfield images of AsPC-1 and AsPC-1+MRC5 spheroid growth. Scale bar = 250 µm. (**C**) Growth kinetics of AsPC-1 and AsPC-1+MRC5 spheroids based on quantitative analysis of the brightfield images. Depicted are the mean ± 95% CI of N = 16–32 from 3 technical repeats. (**D**) Quantification of HGF secretion in the conditioned media (48 h) of MIA PaCa-2, MRC5, and MIA PaCa-2+MRC5 2D monolayer (co-)cultures (grey bars) or 3D spheroid (co-)cultures (black bars). Depicted are the mean ± SEM of N = 4–6 from ≥2 technical repeats. (**E**) Brightfield images of MIA PaCa-2 and MIA PaCa-2+MRC5 spheroid growth. Scale bar = 250 µm. (**F**) Growth kinetics of MIA PaCa-2 and MIA PaCa-2+MRC5 spheroids based on quantitative analysis of the brightfield images. Depicted are the mean ± 95% CI of N = 16–48 from ≥3 technical repeats. Statistical significance is indicated as * *p* < 0.05, ** *p* < 0.01, *** *p* < 0.005.

Analysis of conditioned media obtained from both 2D and 3D (spheroid) cultures confirmed the secretion of HGF by MRC5 fibroblasts, with minimal secretion by AsPC-1 and MIA PaCa-2 cells (Figure 1A,D). Growth analyses revealed a substantial impact of MRC5 cells on spheroid morphology. In both AsPC-1 and MIA PaCa-2 spheroids, the addition of MRC5 fibroblasts initially induced a notable reduction in spheroid area (Figure 1B,E), followed by accelerated spheroid growth in co-cultures (Figure 1C,F). Confocal microscopy analysis of spheroids in which the individual cell populations were separately labeled with cell tracker dyes revealed that the MRC5 fibroblasts were homogeneously intermingled with the AsPC-1 cells, while clustering of the fibroblasts within the spheroids was observed in the MIA PaCa-2+MRC5 spheroids (Figure 1E and Figure S1).

### 2.2. Recapitulating HGF-MET Signaling in Heterotypic PDAC Spheroids

**Figure 2:**
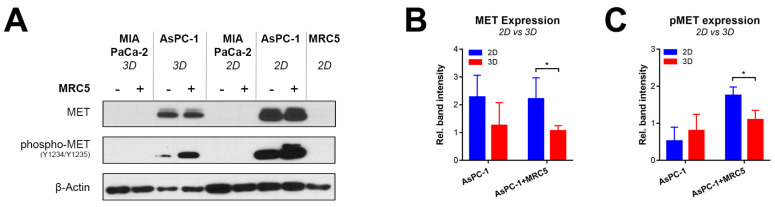
High HGF–MET signaling activity was detected in AsPC-1-based spheroids, but not in MIA PaCa-2-based spheroids. (**A**) Immunoblots depicting the expression levels of MET, phospho-MET (Y1234/1235), and β-actin (loading control) in 2D and 3D cultures composed of AsPC-1, AsPC-1+MRC5, MIA PaCa-2, and MIA PaCa-2+MRC5 cells. (**B**,**C**) Comparative protein band quantification of MET expression (B) and MET phosphorylation (C) in 2D and 3D cultures composed of AsPC-1 and AsPC-1+MRC5 cells. Data represent the mean + SD from four technical repeats and were normalized to their respective loading controls. Statistical significance is indicated as * *p* < 0.05.

We next assessed whether the HGF–MET signaling axis was activated in heterotypic PDAC spheroids. Immunoblotting revealed that the presence of MRC5 caused a substantial increase in MET phosphorylation in 2D and 3D (co-)cultures of AsPC-1 cells, but did not influence overall MET expression levels (Figure 2). Interestingly, AsPC-1 cells exhibited autophosphorylation of the MET receptor even in the absence of MRC5 fibroblasts (Figure 2A). As observed in Figure 1A, AsPC-1 cells secreted low but detectable levels of HGF in both 2D and 3D culture, thus potentially activating the HGF– MET axis through autocrine signaling. Our findings additionally report that, in comparison to 2D cultures, there were substantial reductions in MET expression levels in the 3D spheroid cultures. In contrast, neither MET nor phosphorylated MET was detected in cultures of MIA PaCa-2 cells, both in the absence and presence of MRC5 cells (Figure 2B). Taken together, spheroids composed of AsPC-1 and MRC5 cells demonstrated high activity of the HGF–MET signaling axis, whereas spheroids composed of MIA PaCa-2 and MRC5 cells did not.

### 2.3. PDT Efficacy Is Reduced by the Presence of MRC5 Fibroblasts in PDAC Spheroids

**Figure 3:**
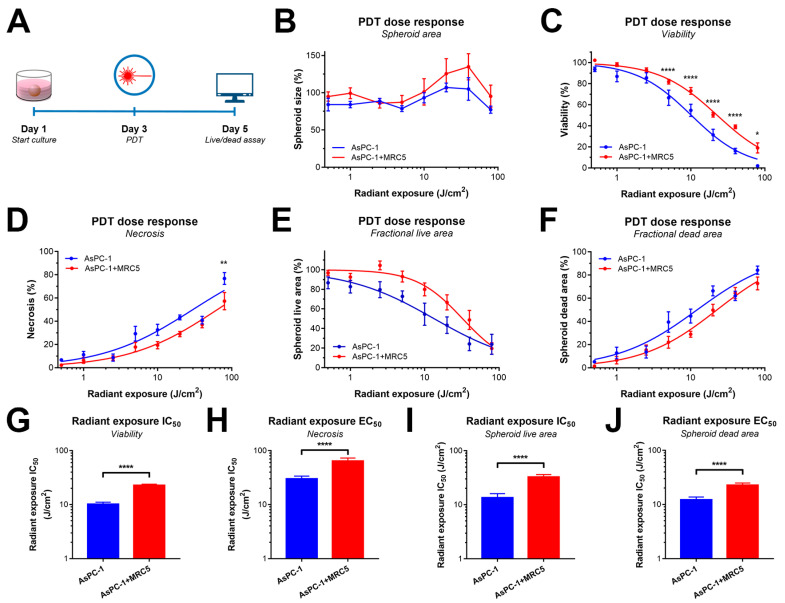
PDT efficacy was reduced by the presence of MRC5 fibroblasts in AsPC-1 spheroids. (**A**) Experimental timeline. (**B**–**F**) PDT dose-response in AsPC-1 (blue) and AsPC-1+MRC5 spheroids (red) based on spheroid viability (B), necrosis (C), spheroid size (D), fractional live area (E), and fractional dead area (F). Data depict the mean ± SEM of N = 12 obtained from three technical repeats. (**G**–**J**) IC_50_ and EC_50_ values were extracted from the dose-response curve fits. Statistical significance is indicated as * *p* < 0.05, ** *p* < 0.01, or **** *p* < 0.001.

We next evaluated whether the presence of MRC5 in AsPC-1 spheroids influenced the efficacy of PDT. In radiant exposure dose-escalation experiments (timeline in Figure 3A), we combined in situ live/dead staining with a recently developed automated image analysis tool for multiparametric assessment of treatment effects [18,26,27]. In this study, we evaluated treatment responses by quantifying viability, necrosis, spheroid area, fractional live area, and fractional dead area.

In AsPC-1+MRC5 spheroids, there was a significant reduction in PDT efficacy compared to spheroids without MRC5 fibroblasts, as reflected in the higher viabilities (Figure 3C,G), and lower necrosis (Figure 3D,H) post-PDT. Analysis of spheroid size did not indicate any dose-dependent effects, indicating that assessing spheroid size alone is not a sufficient means to measure PDT effects (Figure 3B). Fractional live and dead areas also depicted highly significant reductions in PDT efficacy in AsPC-1+MRC5 spheroids (Figure 3E,F,I,J). The fitted IC_50_/EC_50_ values for radiant exposure (Figure 3G–J) differed for the various parameters but uniformly reported a >2-fold reduction in PDT sensitivity for the AsPC-1+MRC5 spheroids compared to AsPC-1 alone.

In MIA PaCa-2+MRC5 spheroids, analysis of PDT efficacies demonstrated a significant reduction in PDT efficacy compared to spheroids composed of MIA PaCa-2 alone (Figure S2). Significant differences between the two culture types were neither observed upon analysis of spheroid necrosis nor upon analysis of the fractional dead area (Figure S2D,F,I,K). In contrast to the AsPC-1 cultures, the MIA PaCa-2-based cultures displayed a clear dose-dependent reduction in spheroid size following PDT (Figure S2B). However, in the presence of MRC5 fibroblasts, the spheroids were less sensitive to this effect and remained more intact following PDT: A substantial, >10-fold reduction in the efficacy of PDT to disrupt spheroid integrity was observed for the MIA PaCa-2+MRC5 cultures compared to MIA PaCa-2 alone (Figure S2G). Analysis of viability and spheroid live area both revealed a significant, 2-fold decrease in PDT efficacy (Figure S2C,H,E,J). Together, these results depict that the integrity of MIA PaCa-2 spheroids is affected by PDT, causing fragmentation into smaller multicellular clusters, consistent with previous observations [17,26,28]. In the presence of MRC5 fibroblasts, these effects appeared to be mitigated. However, these fragmented cell clusters appeared to have comparable viabilities and extents of necrosis that appeared minimally influenced by the presence of MRC5 fibroblasts.

### 2.4. PDT Drives Paracrine MET Signaling by Inducing HGF Secretion by MRC5 Fibroblasts

**Figure 4:**
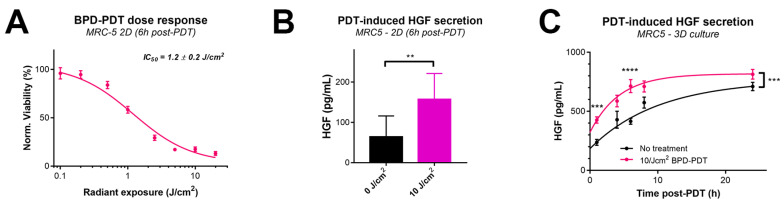
PDT elevates HGF secretion by MRC5 cells in 2D and 3D. (**A**) Efficacy of benzoporphyrin derivative (BPD)–PDT on MRC5 cells cultured in 2D. Depicted are mean ± SEM from N ≥ 12 obtained from four technical repeats. (**B**) Quantification of 10 J/cm^2^ PDT-induced HGF secretion in culture medium by MRC5 cells in 2D (mean ± SD from N = 4–8 from three technical repeats). (**C**) Quantification of HGF secretion in culture media by MRC5 spheroids. Depicted are mean ± SEM from N = 4–23 obtained from four technical repeats. Data were fitted using a one-phase decay equation. Statistical significance is indicated as ** *p* < 0.01, *** *p* < 0.005, or **** *p* < 0.001.

Given the pro-survival function of the HGF–MET axis, we investigated whether this signaling pathway was influenced by PDT. We established a radiant exposure dose-response curve for BPD–PDT in MRC5 fibroblasts cultured in 2D and noted an IC_50_ value of 1.2 ± 0.2 J/cm^2^ (Figure 4A). The curve demonstrates that a radiant exposure of 10 J/cm^2^ reduced MRC5 culture viability to 13.9%, as measured 6 h post-PDT. At the same timepoint and PDT dose, a significant elevation in HGF was detected in the cell culture medium (Figure 4B). Results from two technical repeats revealed that HGF was secreted in a radiant-exposure dose-dependent manner, in which HGF secretion levels plateaued at 10 J/cm^2^ (Figure S3A). There was a linear correlation between PDT-induced cell death and HGF secretion levels from MRC5 fibroblasts (Figure S3B). We next evaluated whether MRC5 fibroblasts cultured in 3D exhibited similar behavior when treated with a dose of 10 J/cm^2^. As expected, there was a time-dependent accumulation of secreted HGF by non-treated MRC5 spheroids; however, HGF secretion was significantly elevated by PDT (Figure 4C). Statistical analysis revealed a significantly elevated rate constant for HGF secretion by MRC5 fibroblasts treated with BPD–PDT compared to untreated fibroblasts (*K* = 0.23 ± 0.09 h^−1^ vs. *K* = 0.10 ± 0.03 h^−1^, *p* = 0.003).

In AsPC-1 spheroids, PDT induced an immediate release of HGF that peaked 4 h post-PDT, which was reduced at later time points (Figure S3C). Heterocellular AsPC-1+MRC5 spheroids demonstrated a delayed release of HGF following PDT, peaking 24 h post-PDT (Figure S3C). However, HGF secretion in AsPC-1 and AsPC-1+MRC5 spheroids was only significantly different 24 h post-PDT. AsPC-1 cells may sequester extracellular HGF through the high expression of MET. This sequestration could influence the extent to which HGF can be accurately detected in these cultures. We, therefore, further explored the impact of PDT on MET signaling. Results from two technical repeats indicated that MET expression was reduced in AsPC-1 and AsPC-1+MRC5 spheroids following PDT (Figure S3D,E). This effect is likely a non-specific event, as a similar decrease in EGFR levels was also detected. We hypothesized that the remaining MET in the AsPC-1+MRC5 spheroids would be activated by the PDT-induced release of HGF from MRC5, but not in the spheroids containing AsPC-1 cells alone. An investigation into phospho-MET levels provided some evidence to support this hypothesis (Figure S3D–F), although inconsistencies in the repeated analyses suggest that it remains to be fully validated. We, thus, set out to evaluate the impact of inhibiting MET signaling on PDT outcomes as an alternative approach to determine the importance of the HFG-MET cascade.

### 2.5. Inhibiting MET Phosphorylation with Cabozantinib Significantly Improves PDT Efficacy

**Figure 5:**
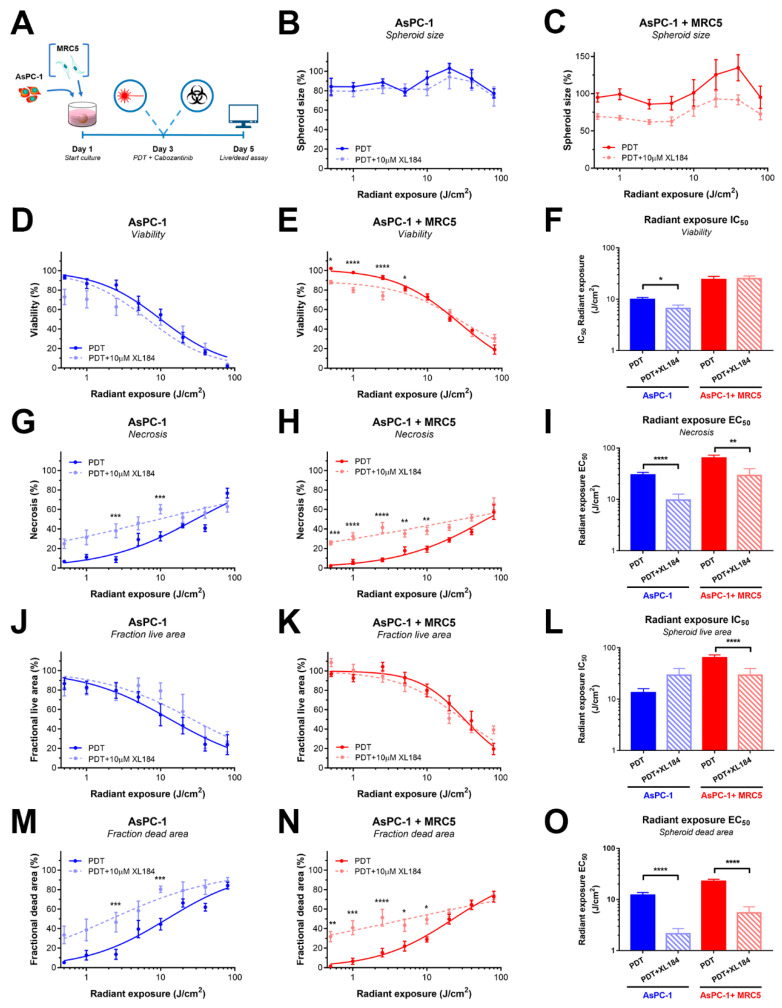
Cabozantinib augments the capacity of PDT to elevate necrosis in AsPC-1 and AsPC-1+MRC5 spheroids. (**A**) Schematic overview of the experimental timeline. (**B**,**C**) Effect of cabozantinib + PDT on overall AsPC-1 (**B**) and AsPC-1+MRC5 (**C**) spheroid size. (**D**–**F**) The effect of cabozantinib on the PDT efficacy based on spheroid viability in AsPC-1 (D), AsPC-1+MRC5 spheroids (E), and a statistical comparison of the fitted IC_50_ values (F). (**G**–**I**) The effect of cabozantinib on the PDT efficacy based on spheroid necrosis in AsPC-1 (G), AsPC-1+MRC5 spheroids (H), and a statistical comparison of the fitted EC_50_ values (**I**). (**J**–**L**) The effect of cabozantinib on the PDT efficacy based on the fractional live area of AsPC-1 (J), and AsPC-1+MRC5 spheroids (K), and a statistical comparison of the fitted IC_50_ values (L). (**M**–**O**) The effect of cabozantinib on the PDT efficacy based on the fractional dead area of AsPC-1 (M), and AsPC-1+MRC5 spheroids (N), and a statistical comparison of the fitted EC_50_ values (O). All data are the mean ± SEM from N = 12 from three technical repeats. The error in IC_50_ values represents the SD. Statistical significance is indicated as * *p* < 0.05, ** *p* < 0.01, *** *p* < 0.005, or **** *p* < 0.001.

To investigate whether the abrogation of the HGF–MET signaling axis could improve PDT outcomes, a combination treatment of PDT and cabozantinib (XL-184) was investigated. To prevent potential oxidative photodestruction of cabozantinib during PDT, the treatment sequence consisted of PDT followed by the addition of cabozantinib. An overview of the experimental timeline is given in Figure 5A. We found that complete inhibition of MET phosphorylation in AsPC-1 spheroid cultures could be achieved with a concentration of 10 µM cabozantinib (Figure S4A). This concentration exerted minor toxicity to both AsPC-1 and AsPC-1+MRC5 spheroids following a 72 h exposure, but had overall poor efficacy as a single therapy (Figure S4B,C). Similar results were obtained in MIA PaCa-2 and MIA PaCa-2+MRC5 spheroids (Figure S4D,E). In all culture types, we noted a minor yet significant decrease in spheroid viability when simultaneously exposed to cabozantinib and BPD in the absence of light exposure. This reduction in viability necessitated the normalization of the data in all subsequent PDT-dose response curves to either the 0 J/cm^2^ controls or the 0 J/cm^2^ + 10 µM cabozantinib controls to accurately determine the effect of cabozantinib on PDT efficacy.

In AsPC-1 spheroids devoid of MRC5 fibroblasts, cabozantinib exerted only a minor yet significant effect on the overall viability of the spheroids (Figure 5D,F), and no significant dose-dependent effects on spheroid size (Figure 5B). A strong increase in spheroid necrosis was observed, translating to a highly reduced EC_50_ value (3-fold reduction, *p* < 0.001, Figure 5G,I). There was no significant effect on the fractional live area of the spheroids (Figure 5J,L), yet a strong increase in the fractional dead area of the spheroids was observed (Figure 5M). Similar to the total spheroid necrosis, this translated to a strongly reduced EC_50_ value (Figure 5O).

Similar trends of enhanced PDT efficacy by neoadjuvant cabozantinib were also observed in AsPC-1+MRC5 spheroids. Although there was no significant improvement detected on overall spheroid size and viability (Figure 5B,E,F), the combination therapy achieved significant increases in spheroid necrosis (Figure 5H,I) and fractional dead spheroid areas (Figure 5N,O). Curve fits report a 2- and 4-fold increase in PDT efficacy, respectively. Interestingly, a significant decrease in the IC_50_ based on the fractional live area was also observed for the combination therapy (Figure 5L), despite that the dose-response curves appear similar (Figure 5K). Statistical comparisons of the individual PDT or PDT+XL-184 treatment groups revealed that the effects of low-dose PDT were particularly amplified by neoadjuvant cabozantinib.

**Figure 6:**
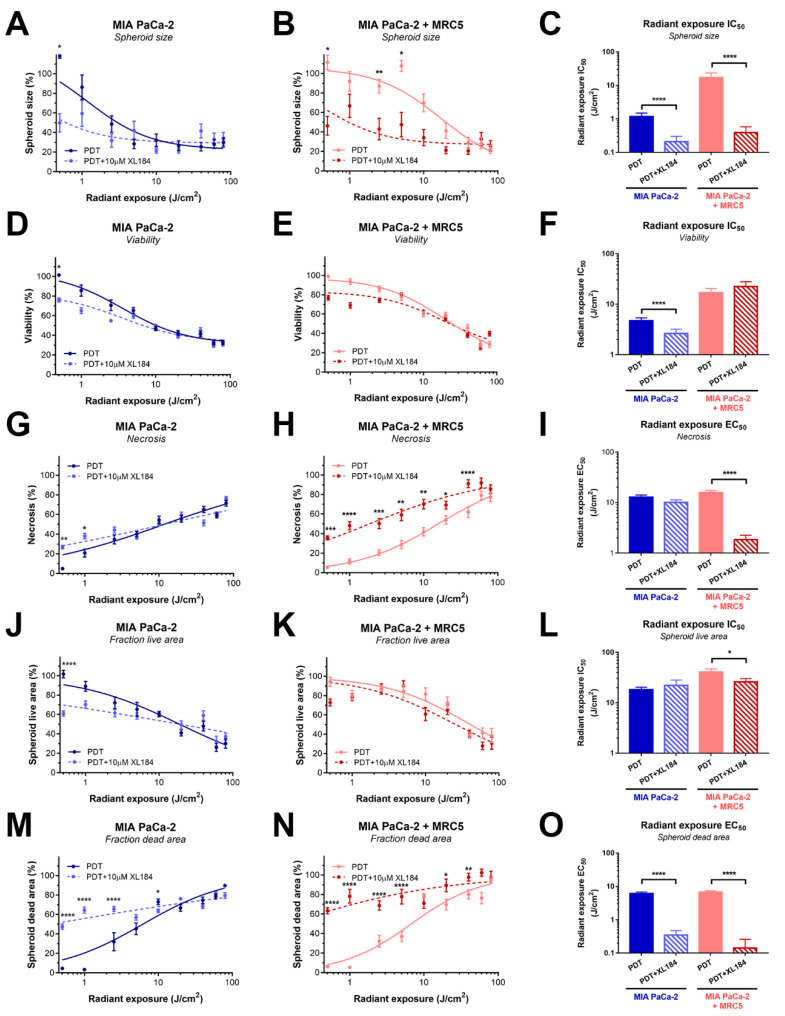
Cabozantinib augments the capacity of PDT to reduce spheroid sizes and elevate necrosis in MIA PaCa-2 and MIA PaCa-2+MRC5 spheroids. (**A**–**C**) The effect of cabozantinib on the PDT efficacy based on the size of MIA PaCa-2 spheroids (A), MIA PaCa-2+MRC5 spheroids (B), and a statistical comparison of the fitted IC_50_ values (C). (**D**–**F**) The effect of cabozantinib on the PDT efficacy based on the viability of MIA PaCa-2 spheroids (D), MIA PaCa-2+MRC5 spheroids (E), and a statistical comparison of the fitted IC_50_ values (F). (**G–I**) The effect of cabozantinib on the PDT efficacy based on overall necrosis in MIA PaCa-2 spheroids (G), MIA PaCa-2+MRC5 spheroid (H), and a statistical comparison of the fitted EC_50_ values (I). (**J–L**) The effect of cabozantinib on the PDT efficacy based on the fractional live area of MIA PaCa-2 spheroids (J), and MIA PaCa-2+MRC5 spheroids (K), and a statistical comparison of the fitted IC_50_ values (L). (**M–O**) The effect of cabozantinib on the PDT efficacy based on the fractional dead area of MIA PaCa-2 (GM), and MIA PaCa-2+MRC5 spheroids (N), and a statistical comparison of the fitted EC_50_ values (O). All data are the mean ± SEM from N = 8–36 from ≥3 technical repeats. The error in IC_50_ values represents the SD. Statistical significance is indicated as * *p* < 0.05, ** *p* < 0.01, *** *p* < 0.005, or **** *p* < 0.001.

In MIA PaCa-2 spheroids, cabozantinib significantly improved the capacity of PDT to reduce overall spheroid area (Figure 6A,C), spheroid viability (Figure 6D,F), and the spheroid fractional dead areas (Figure 6M,O). Despite statically significant elevations in spheroid necrosis and reduction in spheroid live areas at low PDT doses (Figure 6G,J), cabozantinib did not significantly influence the overall dose-response curves based on these parameters (Figure 6I,L).

In MIA PaCa-2+MRC5 spheroids, cabozantinib treatment resulted in strong and statistically significant increases in PDT efficacy, as observed by spheroid area (Figure 6B,C), necrosis (Figure 6H,I), and fractional dead area (Figure 6N,O). A minor improvement was observed based on the overall reductions in spheroid live area (Figure 6K,L), yet no effect was observed based on spheroid viability (Figure 6E,F). Similar to the AsPC-1-based cultures, it appeared that cabozantinib was most effective at elevating the efficacy of low-dose PDT.

Overall, the findings convincingly demonstrate a substantial improvement in BPD–PDT efficacy when combined with cabozantinib. The treatment effects occurred in different manners in the varying spheroid types. AsPC-1-based spheroids mostly displayed elevated levels of spheroid necrosis, whereas MIA PaCa-2-based spheroids additionally exhibited strongly reduced spheroid sizes. Cabozantinib was most effective in combination with low-dose (<10 J/cm^2^) PDT regimens. This is likely related to the eradication of the MRC5 fibroblasts at higher doses (Figure 4A), where paracrine HGF signaling may no longer be stimulated by these cells.

## 3. Discussion

The current study aimed to obtain detailed mechanistic insights into the role of the HGF–MET axis in mediating PDT resistance and its rational inhibition by cabozantinib to mitigate resistance. To do so, heterotypic pancreatic cancer spheroids comprising both PDAC cell lines and HGF-secreting fibroblasts were established. However, spheroids composed of AsPC-1 cells alone showed that MET signaling can be auto-activated even in the absence of HGF-secreting fibroblasts. PDT was shown to induce the release of HGF from dying fibroblasts and may play a significant role in facilitating sustained MET survival signaling. Cabozantinib was shown to be capable of abolishing MET phosphorylation and enhancing the efficacy of PDT in 3D PDAC microtumors regardless of MET expression levels. Cabozantinib significantly increased efficacy of PDT, most notably in the low radiant exposure (0.5–10 J/cm^2^) range. These results underscore the potential of this combination therapy, which can yield beneficial outcomes regardless of MET expression levels in PDAC tumors.

A variety of molecular signaling pathways have been identified in the adaptive responses of cancer cells that enable cells to cope with the effects of PDT [13]. These are mostly intrinsic pathways that implicate the transcription factors nuclear factor (erythroid-derived 2)-like 2 (NRF2), hypoxia-inducible factor 1 (HIF-1), nuclear factor-κB, Activator protein 1 (AP-1), and heat shock factors (HSFs) in mediating reduced sensitivity to PDT [13,29,30,31,32,33,34,35,36]. The results of this study clearly identified MET signaling as an additional auto- and paracrine survival pathway in cancer cells that can be induced and sustained by HGF secretion from perishing stromal cells. HGF is a mitogenic glycoprotein that is secreted primarily by fibroblasts [37], and binds MET that is expressed almost exclusively by epithelial cells [38]. Subsequent prolonged MET signaling following PDT may promote cell motility, proliferation, stem cell maintenance, and therapy resistance in cancer cells of epithelial origin [39], potentially resulting in an overall decrease in therapeutic efficacy. Its clinical relevance to pancreatic cancer has also been demonstrated, with various studies reporting on the overexpression of MET in pancreatic cancers [40] and a correlation between elevated serum levels of HGF and aggressive PDAC phenotypes [41]. These reports underscore that the HGF–MET signaling axis is a promising therapeutic target to overcome treatment resistance in PDAC.

A study by Cuneo et al. identified that notable MET expression was detected in 86% of PDAC patients, with abnormally high expression levels noted in 64% of the total patient population [42]. Since high MET expression is associated with greatly reduced progression-free survival [42], cabozantinib appears to be a viable therapeutic candidate for PDAC. Despite promising preclinical data, the high dose-limiting toxicity for cabozantinib restricts its further clinical use for PDAC patients [10]. In contrast, toxicity profiles have been deemed acceptable for the treatment of patients with hepatocellular carcinoma, and clinical trials have yielded promising results regarding the use of cabozantinib for this disease [43]. When comparing these studies, the patients with hepatocellular carcinoma received a daily dose of 60 mg, whereas the PDAC patients received 20–40 mg per day. All patients with hepatocellular carcinoma had received sorafenib chemotherapy prior to the trial but received no additional treatment during the trial. In contrast, the advanced PDAC patients continued to receive gemcitabine during the trial. The diverging toxicity profiles may be related to the combination with gemcitabine, but further studies are necessary to provide conclusive evidence. In our study, we noted that BPD and cabozantinib had negligible (dark) toxicity when used as monotherapies at 0.25 and 10 µM, respectively, but had notable (dark) toxicity when these agents were combined. The exact nature of this toxicity warrants further investigation. Our findings, both presented here and previously [24], provide strong indications that PDT can aid in making cabozantinib more effective, and thus reduce the dose-limiting toxicities of this multi-kinase inhibitor. Other multi-kinase inhibitors have also been shown to be effective in augmenting the anti-tumor PDT effect in vivo. Inhibiting EGFR and HER-2 simultaneously using a liposomal formulation of lapatinib resulted in enhanced amino-levulinic acid-PDT efficacies and prolonged survival in an orthotopic rat model of glioma [44].

Heterotypic 3D models of PDAC that include multiple cell types are valuable platforms for preclinical treatment screening as they can recapitulate stromal cues that influence treatment efficacy. This study, and others, have demonstrated that fibroblasts, including cancer-associated fibroblasts and pancreatic stellate cells, induce notable changes in spheroid/organoid morphology and growth, PDAC metabolism, and treatment susceptibility [23,27,45,46,47,48,49,50]. This study demonstrates the additional capacity of these models to recapitulate specific paracrine signaling events, such as the HGF–MET axis, although the cell lines were carefully selected to represent this specific signaling axis. In agreement with previous studies [24], cabozantinib proved effective in prohibiting MET phosphorylation and exerted an additive effect on PDT efficacy. The significant increases in PDT efficacy in 3D heterocellular cultures observed here does not fully recapitulate the promising effects observed in vivo, in which a combination of PDT and cabozantinib greatly enhanced tumor regression and reduced metastatic burdens [24]. Interestingly, these effects were observed in both AsPC-1 and MIA PaCa-2 tumors that were orthotopically implanted, despite the absence of MET expression by the MIA PaCa-2 cell lines. Therefore, the current findings suggest that the mesoscopic treatment effects of cabozantinib, such as impaired angiogenesis by VEGFR2 inhibition, are major contributors to the overall treatment efficacy that can currently not be fully recapitulated using heterotypic 3D cultures in vitro. In addition, the in vivo impact of HGF–MET signaling on angiogenesis and treatment resistance may provide further insights into the potentiating effect that cabozantinib has on PDT in vivo [51]. Future work using heterotypic desmoplastic organoids that recapitulate the vascularization of tumors may offer further understanding of the mechanisms of synergistic anti-tumor combinations, such as PDT with cabozantinib. Auxiliary inhibition of treatment escape by cabozantinib may also stem from impaired AXL and RET activity [52], although further research is needed to identify the therapeutic contributions of these effects.

## 4. Materials and Methods

### 4.1. Cell Culture

All cell lines were obtained from the American Type Culture Collection (Manassas, VA, USA). AsPC-1 cells were maintained in RPMI medium (Corning, Corning, NY, USA), MIA PaCa-2 were maintained in DMEM medium (Corning), and MRC5 cells were maintained in MEM medium (Corning). All types of culture media were supplemented with 1% (v/v) penicillin/streptomycin (Corning) and 10% (v/v) fetal bovine serum (FBS, Gibco, ThermoFisher, Waltham MA, USA). Culture media were changed every 3–4 days, and cells were passaged weekly. Cells were discarded after passage 25 and tested negative for mycoplasma.

AsPC-1, MIA PaCa-2, and MRC5 cells were seeded at a density of 5000 cells per well per cell line in ultra-low attachment round-bottom plates (Corning) in a final volume of 100 µL in a 1:1 growth media mixture. Cells were either seeded as a single cell type (5000 cells/well) or a mixture of either AsPC-1 or MIA PaCa-2 cells, combined with MRC5 fibroblasts (5000 + 5000 cells/well). When two cell lines were mixed, they were added within 30 min of each other to ensure heterogeneous distribution.

Spheroid growth was determined by daily brightfield imaging (Operetta CLS, Perkin–Elmer, Billerica, MA, USA). Image acquisition was performed using a 5× air objective (0.16 N.A.), at a resolution of 1080 × 1080 px^2^. From the brightfield images, spheroid areas were extracted using a custom-written script in MATLAB 2016b (Mathworks, Natick, MA, USA).

### 4.2. Tracking of Cell Populations in Spheroid Cultures

AsPC-1, MIA PaCa-2, and MRC5 were seeded separately in 6-well plates at a density of 1 × 10^6^ cells per well and were allowed to attach for 24 h. Culture media were removed, and cells were washed twice with phosphate buffer saline (PBS). A PBS solution containing 5 µM of CellTracker Red CMTPX (#C34552, Molecular probes, Thermo Fisher) or 5 µM CellTracker Deep Red (#C34565, Molecular probes) was added to AsPC-1/MIA PaCa-2 or MRC5, respectively, and cells were incubated for 45 min at 37 °C. The solution was aspirated, and labeled cells were harvested by trypsinization. Labeled cells were then used for spheroid culture initiation, as described above. Images were taken on culture day 3 using an Olympus FV1000 confocal laser scanning microscope (10× air objective, 0.4 N.A.). Image acquisition parameters were λ_exc_ = 559 nm, λ_em_ = 580 ± 50 nm (CellTracker Red) and λ_exc_ = 635 nm, λ_em_ = 750 ± 50 nm (CellTracker Deep Red). The image resolution was set up at 1024 × 1024 px^2^.

### 4.3. Immunoblotting

Total protein lysates were collected from 2D monocultures or spheroids using RIPA buffer (Thermo Scientific) containing 1% protease inhibitor cocktail (Calbiochem, Merck Millipore, Danvers MA, USA) and 1% phosphatase inhibitors 2 and 3 (Sigma–Aldrich, St. Louis MO, USA). Lysates were centrifuged at 16,000 rpm at 4 °C to remove cellular debris and then transferred into a sterile 1.5 mL tube. Samples of 1–5 µg of protein were separated on 4–20% Tris-HCL gels (Bio-Rad, Hercules CA, USA). Primary antibodies rabbit-anti-MET (clone D1C2), rabbit-anti-phospho-MET (Y1234-Y1235, clone D26), and rabbit-anti-β-actin (clone 13E5) were purchased from Cell Signaling Technologies (Danvers, MA, USA) and were used at 1:1000 dilution ratios. All appropriate horseradish peroxidase-conjugated secondary antibodies were also purchased from Cell Signaling Technologies and used at 1:2000 dilution ratios.

### 4.4. Enzyme-Linked Immunosorbent Assay

AsPC-1, MIA PaCa-2, and MRC5 were cultured as monolayers in a 6-well plate at a density of 1.5 × 10^5^ cells per well, or as spheroid cultures, as described above. Conditioned culture media were collected after 48 h of culturing. Pro-HGF levels were measured using an ELISA (R&D Systems, Minneapolis, MN, USA) using 50 µL of culture medium per sample. We ensured that signal collection never exceeded the upper detection limit of the assay. In experiments, when HGF secretion was measured following PDT in monolayer cultures, MRC5 cells were seeded in black-walled, flat-bottom 96-well plates at a density of 1000 cells per well. The culture medium was harvested after various time intervals, as indicated.

### 4.5. Treatments

PDT was initiated on culture day 3. A 3× stock solution containing 0.75 µM benzoporphyrin derivative (BPD, US Pharmacopeia) was prepared in full growth media and added directly to each well to reach a final concentration of 0.25 µM. After 1 h incubation, 100 µL of culture media containing BPD was carefully aspirated and gently replaced with 100 µL of full growth media. A custom-made black insert that prevented light scattering was used to ensure correct PDT dosimetry in each well. Spheroids or cells were irradiated with 150 mW/cm^2^, 690 nm laser light (Intense, North Brunswick, NJ, USA) at cumulative radiant exposures ranging from 0–80 J/cm^2^ as indicated. After PDT, all cultures were maintained at standard culture conditions.

Cabozantinib (XL-184, Selleckchem, Houston TX, USA) was dissolved in cell culture-grade DMSO (Sigma-Aldrich). A 3× stock solution of 30 µM XL-184 was prepared in full growth media and then filter-sterilized. For 3D cultures, each well containing 100 µL of media received 50 µL of stock solution to yield a final concentration of 10 µM XL-184. XL-184 was not present when cells were irradiated for PDT to prevent its oxidation but was added immediately after PDT from culture days 3–5, as indicated in the treatment timelines.

### 4.6. Multiparametric Analysis of Treatment Response

Treatment efficacy was determined by in situ Live/Dead staining using calcein AM (ThermoFisher) and propidium iodide (Sigma–Aldrich), followed by fluorescence imaging (Olympus FV1000 confocal laser scanning microscope), as optimized for 3D cultures [26]. Derivation of outcome parameters was done using quantitative image analysis according to the CALYPSO methodology, as described previously [27]. When indicated, culture viability was assessed based on NAD(P)H oxidase activity using a CellTiter 96 aqueous non-radioactive proliferation assay (MTS, Promega, Madison, WI, USA), which was performed in accordance with the manufacturer’s recommendation.

### 4.7. Statistical Analysis

All statistical analyses were performed in Prism 5.0 (Graphpad, La Jolla, CA, USA). Growth curves were fitted using a Gompertz growth equation. Data were tested for normality using the Shapiro–Wilk method, after which the data were statistically compared using a One-way ANOVA and Sidak’s post-hoc test (normally distributed data), or using a Kruskal–Wallis and Dunn’s post-hoc test (non-normal data or data with N < 4). Dose response curves were obtained using standard inhibitor/agonist versus normalized response fits, and IC_50_/EC_50_ values were compared using extra sum-of-squares f-tests. Statistically significant differences between treatment groups are indicated with either a single asterisk (*p* ≤ 0.05), double asterisk (*p* ≤ 0.01), triple asterisk (*p* ≤ 0.005), or a quadruple asterisk (*p* ≤ 0.001).

## 5. Conclusions

Modulating the cancer stroma has been considered a potentially effective approach for pancreatic cancer treatment [23,53]. We demonstrated that in a stroma-rich spheroid model that recapitulates the HGF–MET signaling pathway, a combination treatment that incorporates cabozantinib provides a significant additive effect on the therapeutic efficacy of PDT. The findings underscore the necessity of understanding the stromal cues that promote treatment resistance in PDAC and highlight the potential to therapeutically target these intercellular signaling pathways that are specific to desmoplastic cancers. The potentially high toxicity of such therapeutic regimens may be mitigated by developing PDT as an adjuvant therapy, as exemplified here for cabozantinib.

## Supplementary Materials

The following are available online at https://www.mdpi.com/2072-6694/12/6/1401/s1, Figure S1: Cell tracker imaging informs on the dispersion of the MRC5 fibroblasts in spheroid co-cultures, Figure S2: PDT efficacy is reduced by the presence of MRC5 fibroblasts in MIA PaCa-2 spheroids, Figure S3: Impact of PDT on HGF secretion by MRC5 fibroblasts and MET expression and phosphorylation in AsPC-1 spheroids, Figure S4: Toxicity evaluation of 10 µM cabozantinib on heterotypic PDAC spheroids.
