# Supplementary material for: Cabozantinib Inhibits Photodynamic Therapy-Induced Auto- and Paracrine MET Signaling in Heterotypic Pancreatic Microtumors
Source: Cancers (Basel). 2020 May 29;12(6):1401. doi: 10.3390/cancers12061401 (PMC7352584; doi:10.3390/cancers12061401)
Supplement: Supplementary file 1 [file cancers-12-01401-s001.zip › cancers-814539-supplementary checked .docx]

Article

Cabozantinib Inhibits Photodynamic Therapy-Induced Auto- and Paracrine MET Signaling in Heterotypic Pancreatic Microtumors

Mans Broekgaarden, Ahmed Alkhateeb, Shazia Bano, Anne-Laure Bulin, Girgis Obaid, Imran Rizvi and Tayyaba Hasan

1. Supplemental Figures


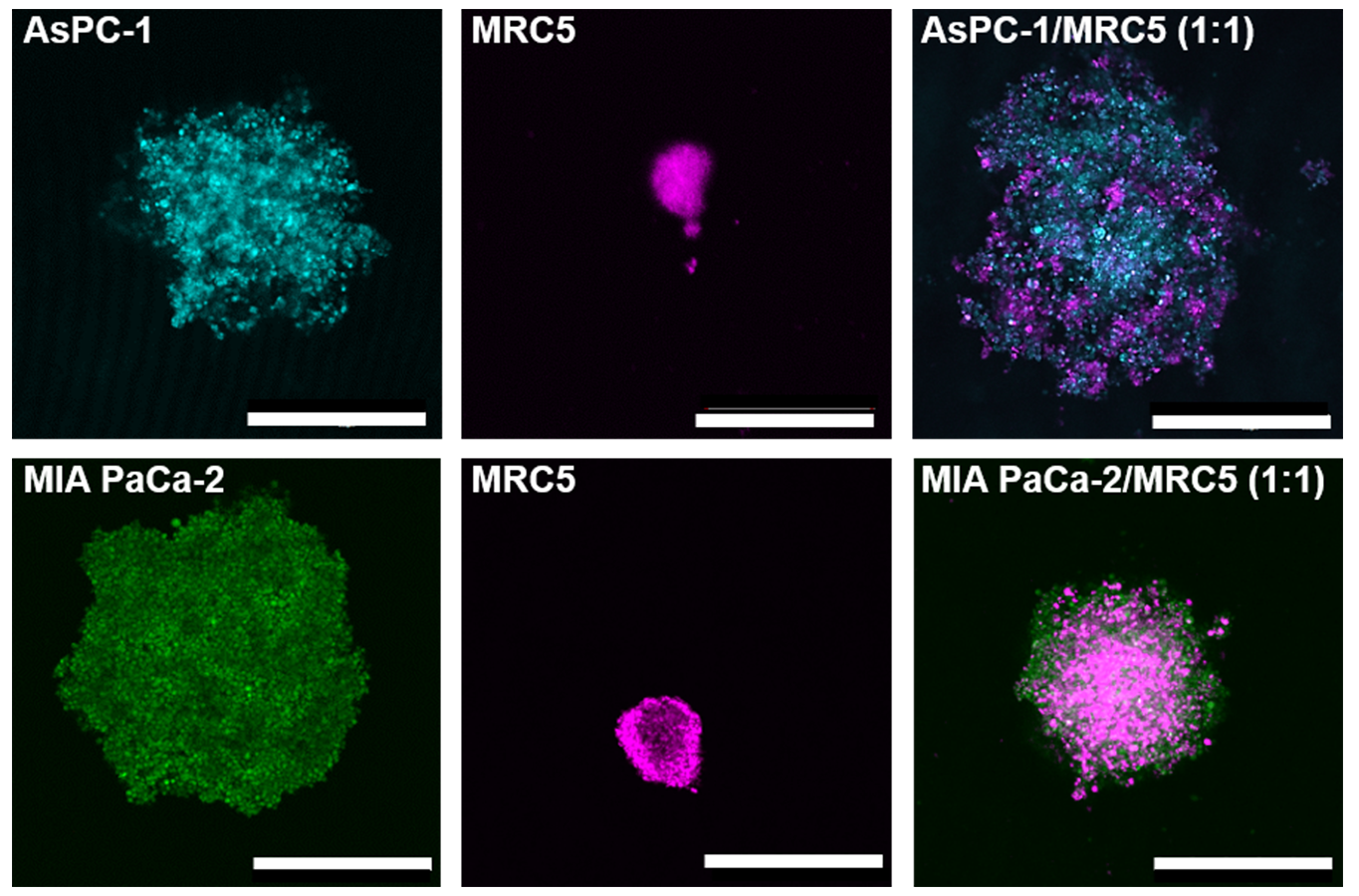


**Figure S1.** Cell tracker imaging informs on the dispersion of the MRC5 fibroblasts in spheroid co-cultures. Confocal laser scanning microscopy of AsPC-1, MRC5, and AsPC-1+MRC5 spheroids (upper panels), as well as MIA PaCa-3, MRC5, and MIA PaCa-2+MRC5 spheroids (bottom panels). AsPC-1 and MIA PaCa-2 cells were labeled with Cell Tracker Red (indicated in false color as turquoise and green, respectively), MRC5 with Cell Tracker Deep-red (indicated as purple). Scalebar = 500 µm.


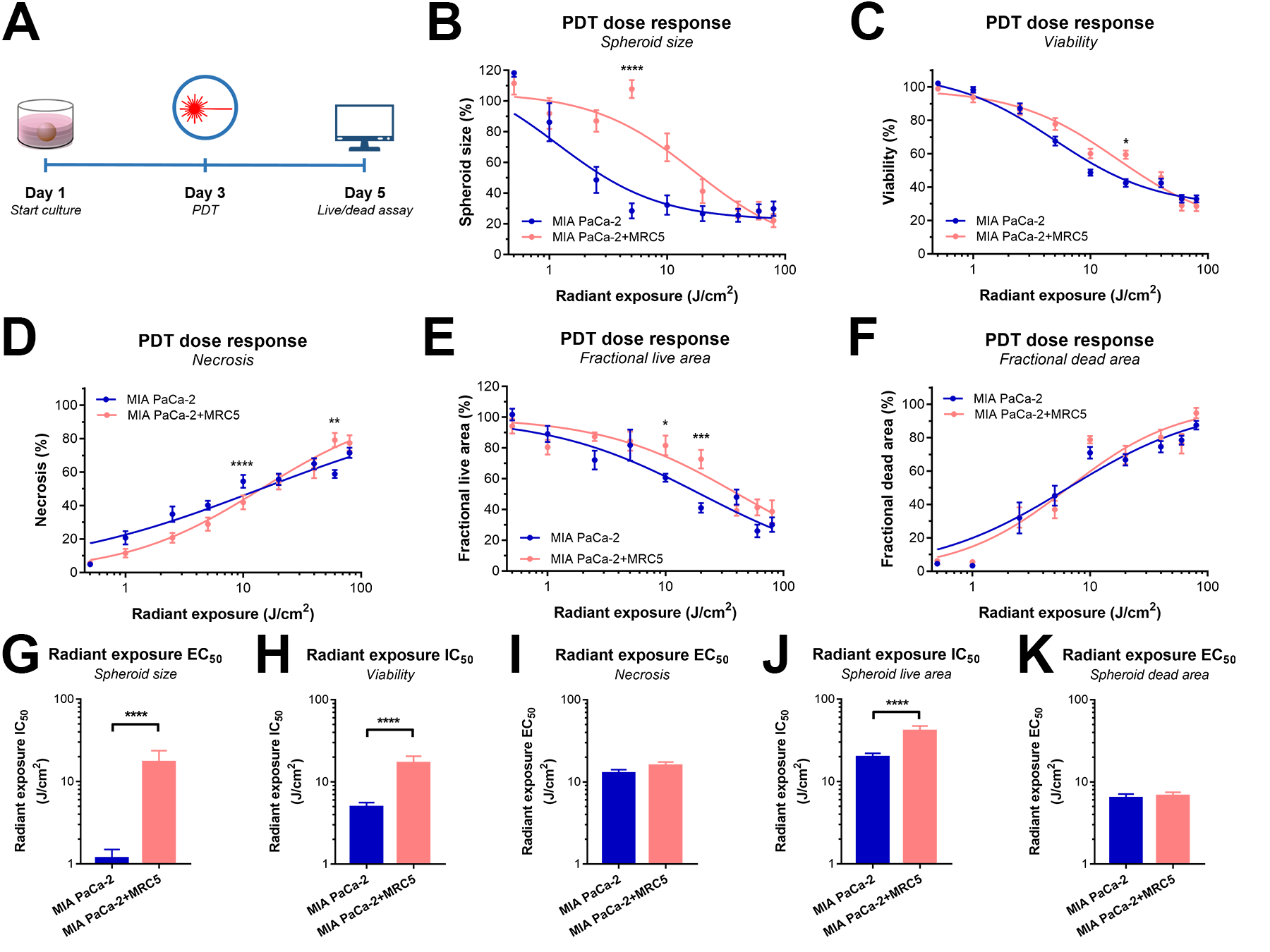


**Figure S2.** PDT efficacy is reduced by the presence of MRC5 fibroblasts in MIA PaCa-2 spheroids. (**A**) Experimental timeline. (**B–F**) PDT dose response in MIA PaCa-2 (dark blue) and MIA PaCa-2+MRC5 (light red) spheroids based on spheroid viability (**B**), necrosis (**C**), spheroid size (**D**), fractional live area (**E**) and fractional dead area (**F**). Data depicts the mean ± SEM of N = 12 obtained from 3 technical repeats. **(G–J)** IC_50_ and EC_50_ values were extracted from the dose-response curve fits. *


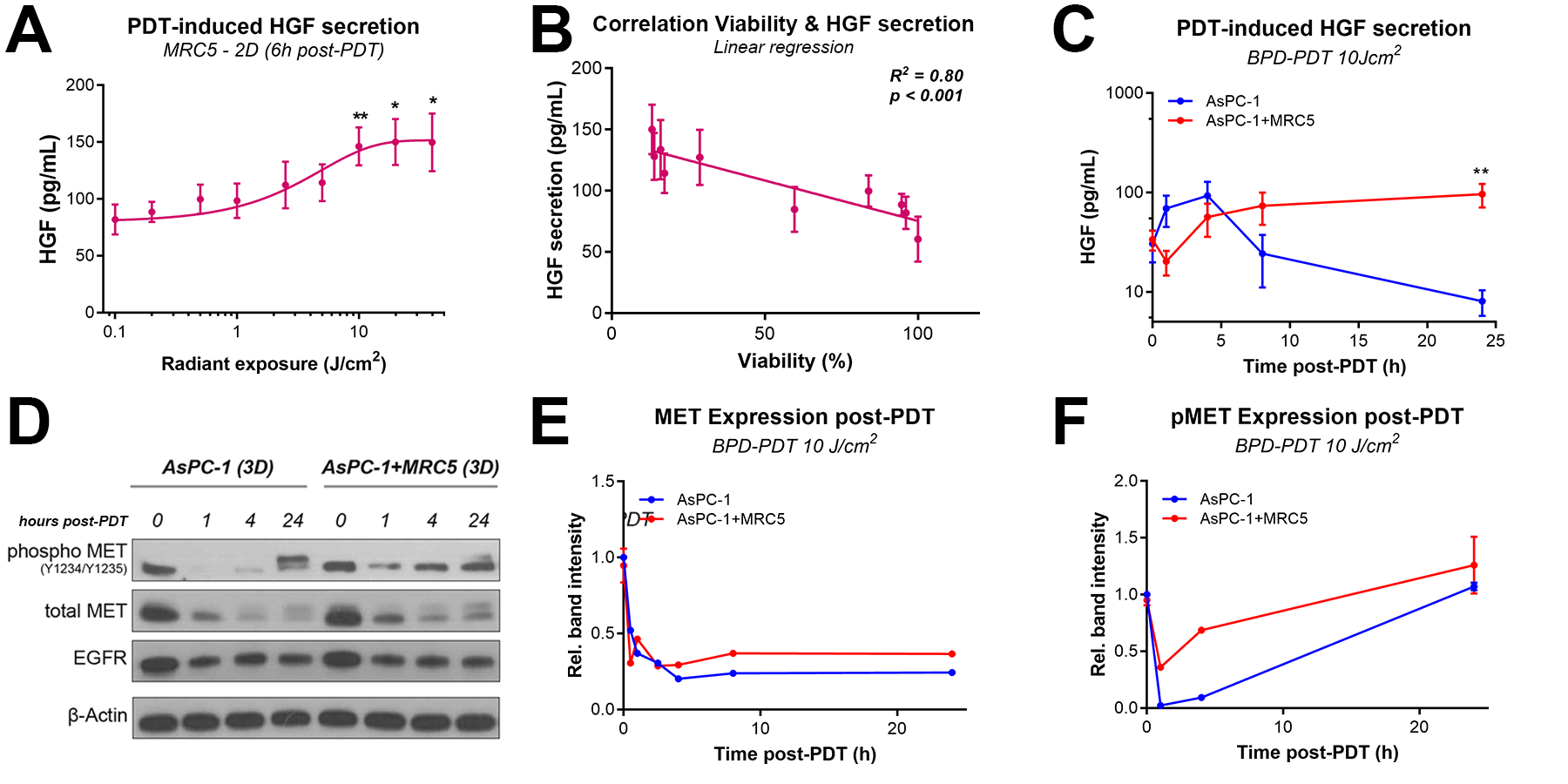


**Figure S3.** Impact of PDT on HGF secretion by MRC5 fibroblast and MET expression and phosphorylation in AsPC-1 spheroids. (**A**) Quantification of PDT-induced HGF secretion in culture medium by MRC5 monolayer cultures (mean ± SEM from N = 4-12 from ≥2 technical repeats). (**B**) Linear regression curve of HGF secretion plotted as a function of the MRC5 viability post-PDT. (**C**) Quantification of HGF secretion in culture medium by AsPC-1 spheroids and AsPC-1+MRC5 spheroids. Depicted are mean ± SEM from N = 6–14 obtained from 4 technical repeats. (**D**) Representative immunoblots depicting the modulation of MET, phospho-MET, EGFR, and β-actin levels following PDT in AsPC-1 and AsPC‑1+MRC5 spheroids. (**E**) Expression levels of MET following PDT in AsPC-1 and AsPC-1+MRC5 cultures (integrated data from 2 separate experiments). (**F**) Quantification of phospho-MET expression levels in AsPC-1 and AsPC-1+MRC5 spheroids following PDT (integrated data from 2 separate experiments).


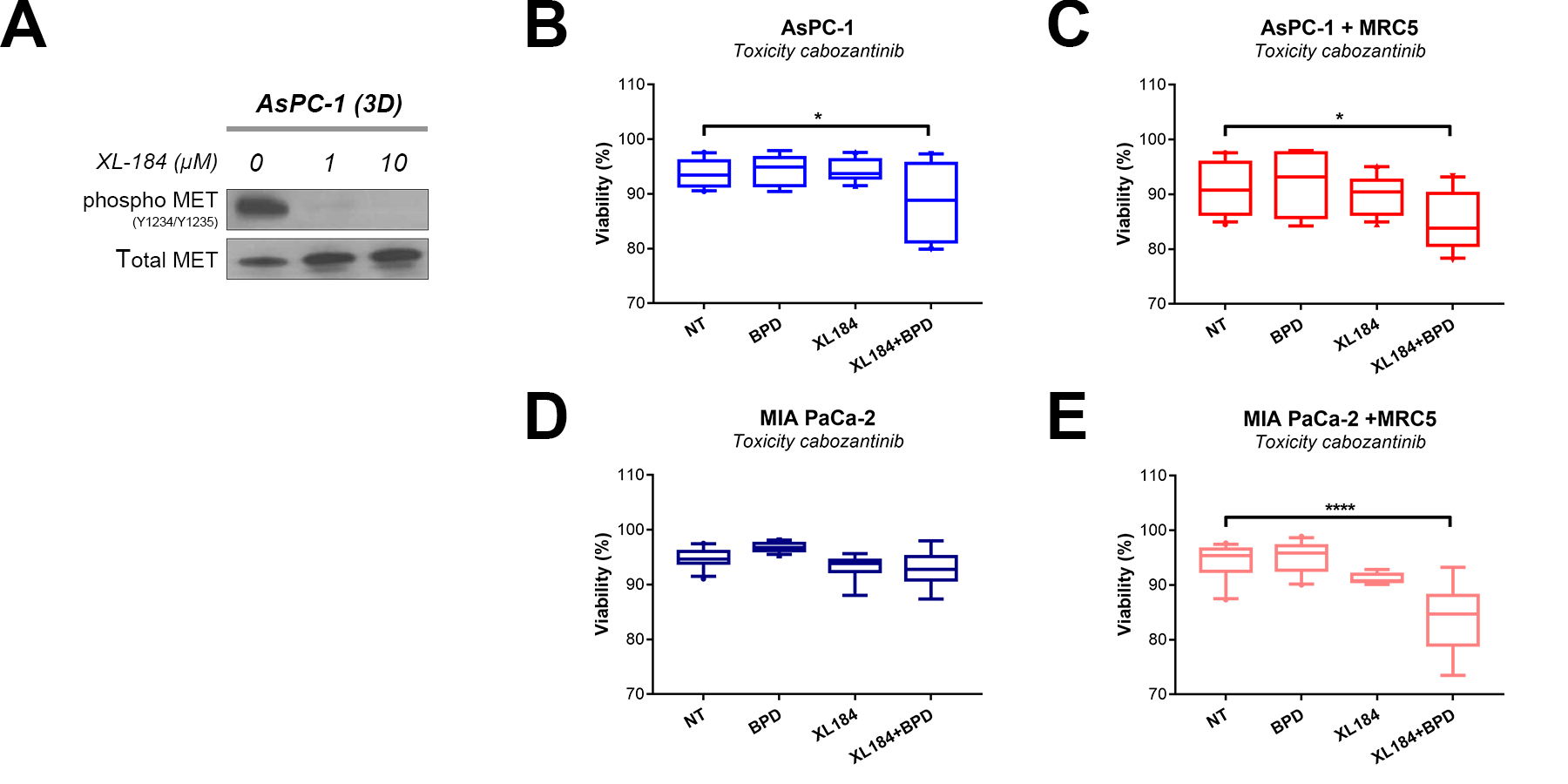


**Figure S4.** Toxicity evaluation of 10 µM cabozantinib on heterotypic PDAC spheroids**.** (**A**) Inhibition of MET phosphorylation by increasing concentrations of cabozantinib (XL-184) on AsPC-1 spheroids (24h incubation). (**B,C**) Toxicity evaluation of 10 µM cabozantinib, 0.25 µM BPD, and 10 µM cabozantinib + 0.25 µM BPD in absence of PDT on AsPC-1 (**B**) and AsPC-1+MRC5 spheroids (**C**) following 72h incubation. **(D,E)** Toxicity evaluation of 10 µM cabozantinib, 0.25 µM BPD, and 10 μM cabozantinib + 0.25 µM BPD in the absence of PDT on MIA PaCa‑2 (**D**) and MIA PaCa-2+MRC5 spheroids (**E**) following 72 h incubation. Data points were from N = 12, obtained from 3 technical repeats.
