# Supplementary material for: Cabozantinib Inhibits Photodynamic Therapy-Induced Auto- and Paracrine MET Signaling in Heterotypic Pancreatic Microtumors
Source: Cancers (Basel). 2020 May 29;12(6):1401. doi: 10.3390/cancers12061401 (PMC7352584; doi:10.3390/cancers12061401)
Supplement: Supplementary file 1 [file cancers-12-01401-s001.zip › Supplement - BroekgaardenCancers2019 - Western Blot and Densitometry.pdf]

Western Blot – Figure 2

Experimental repeat 1

Samples prepared on:
28 March, 2016

1. Ladder	
2. MIA PaCa-2	3D
3. MIA PaCa-2+MRC5	3D
4. AsPC-1	3D
5. AsPC-1+MRC5	3D
6. MIA PaCa-2	2D
7. MIA PaCa-2+MRC5	2D
8. AsPC-1	2D
9. AsPC-1+MRC5	2D
10. MRC5	2D (Not Quantified)

Total MET

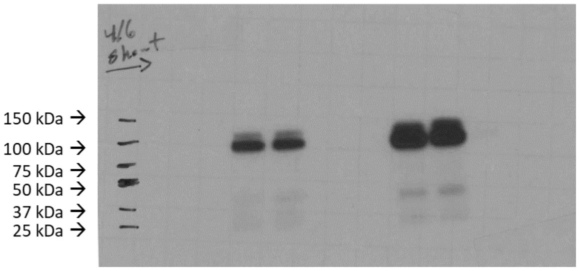

Phospho-MET

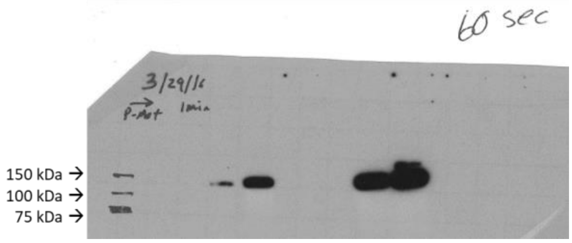

β-Actin

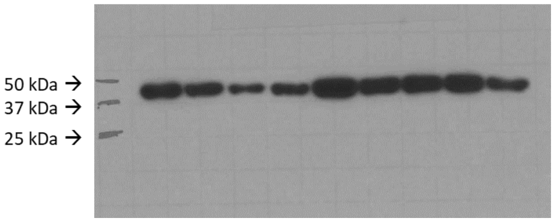

Experimental repeat 2-4

Samples prepared on:
May 28, 2016

Blot 1 (Exp. Repeat 2 & 3)

1. Ladder	
2. AsPC-1 (2)	3D
3. AsPC-1+ MRC5 (2)	3D
4. AsPC-1 (3)	3D
5. AsPC-1+ MRC5 3)	3D
6. Ladder	
7. AsPC-1 (2)	2D
8. AsPC-1+MRC5 (2)	2D
9. AsPC-1 (3)	2D
10. AsPC-1+MRC5 (3)	2D

Total MET (mirrored)

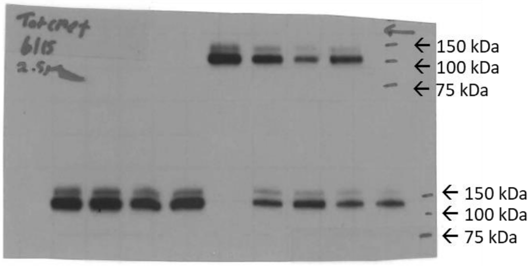

β-Actin (mirrored)

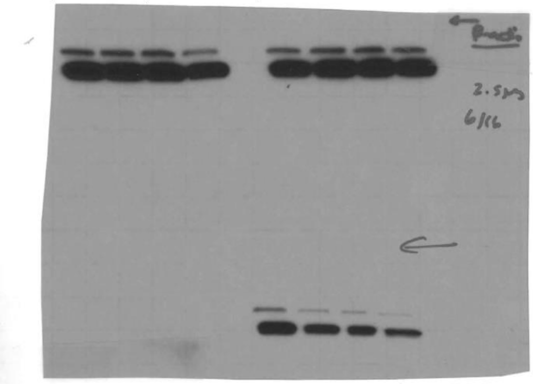

Samples prepared on:
June 2, 2016

Blot 2 (Exp. Repeat 4)

1. Ladder	
2. Ladder	
2. AsPC-1 (4)	3D
3. AsPC-1+ MRC5 (4)	3D
4. AsPC-1 (4)	2D
5. AsPC-1+ MRC5 (4)	2D

Phospho-MET

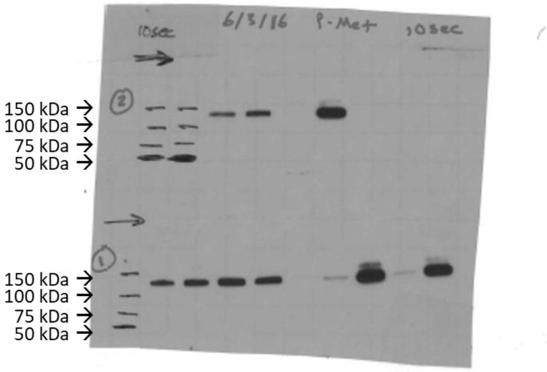

Western Blot – Figure 2

Densitometry

Cell line(s)	Culture type	Exp. Repeat	β-actin	MET	phospho-MET
**MIA PaCa-2**	2D	1	22746.602	47.95	54.778
**MIA PaCa-2+MRC5**	2D	1	18713.409	65.364	98.192
**AsPC-1**	2D	1	11307.075	17851.258	3330.012
**AsPC-1+MRC5**	2D	1	15457.56	17203.844	16559.782
**MIA PaCa-2**	3D	1	30337.794	73.364	77.192
**MIA PaCa-2+MRC5**	3D	1	24556.823	89.364	63.364
**AsPC-1**	3D	1	25398.066	33791.179	26495.459
**AsPC-1+MRC5**	3D	1	25373.723	33106.693	38345.238
**AsPC-1**	3D	2	28880.401	7450.539	10954.104
**AsPC-1+MRC5**	3D	2	32589.279	8480.803	14222.56
**AsPC-1**	3D	3	32602.643	13087.338	15076.125
**AsPC-1+MRC5**	3D	3	26900.501	10576.409	16452.388
**AsPC-1**	2D	2	25411.208	20125.752	7193.619
**AsPC-1+MRC5**	2D	2	32525.572	19457.773	29080.723
**AsPC-1**	2D	3	33164.765	24394.673	6878.912
**AsPC-1+MRC5**	2D	3	30323.907	24222.894	26361.773
**AsPC-1**	3D	4	8980.347	10370.439	10323.326
**AsPC-1+MRC5**	3D	4	10062.175	5385.711	12162.468
**AsPC-1**	2D	4	12402.761	13483.459	723.456
**AsPC-1+MRC5**	2D	4	16739.246	20078.116	26485.288

Western Blot – Figure 4

Experimental repeat 1

Samples prepared on:
3 February, 2016

1. Ladder
2. AsPC-1 — 0h post-PDT
3. AsPC-1 — 0.5h post-PDT
4. AsPC-1 — 2.5h post-PDT
5. AsPC-1 — 8h post-PDT
6. AsPC-1+MRC5 — 0h post-PDT
7. AsPC-1+MRC5 — 0.5h post-PDT
8. AsPC-1+MRC5 — 2.5h post-PDT
9. AsPC-1+MRC5 — 5h post-PDT(not quantif.)
10. AsPC-1+MRC5 — 8h post-PDT

Total MET (mirrored)

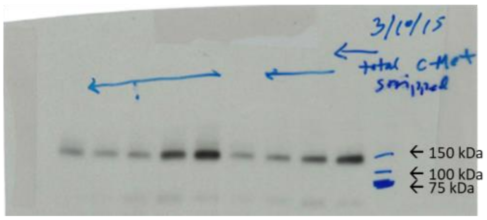

β-actin (mirrored)

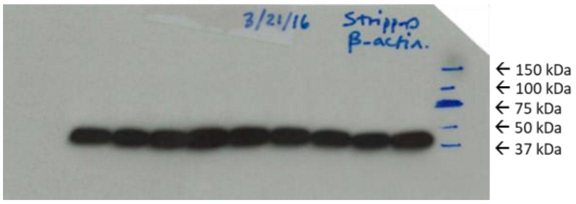

Experimental repeat 2

Samples prepared on:
11 April, 2016

1. Ladder
2. AsPC-1 — 0h post-PDT
3. AsPC-1 — 1h post-PDT
4. AsPC-1 — 4h post-PDT
5. AsPC-1 — 24h post-PDT
6. AsPC-1+MRC5 — 0h post-PDT
7. AsPC-1+MRC5 — 1h post-PDT
8. AsPC-1+MRC5 — 4h post-PDT
9. AsPC-1+MRC5 — 24h post-PDT
10. AsPC-1+MRC5 — 48h post-PDT (not quantif.)

Total MET (mirrored)

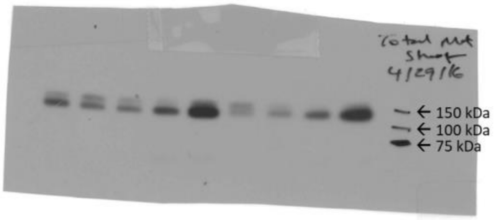

Phospho-MET

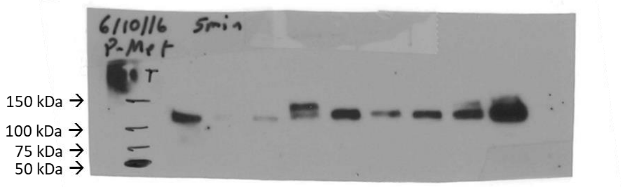

B-actin

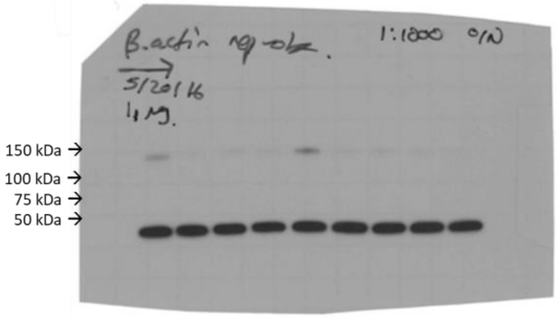

EGFR

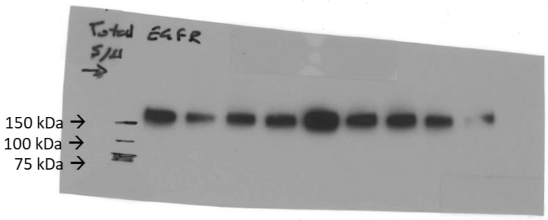

Experimental repeat 3

Samples prepared on:
July 21, 2016

1. Ladder
2. AsPC-1+MRC5 — 0h post-PDT
3. AsPC-1 — 0h post-PDT
4. AsPC-1+MRC5 — 24h post-PDT
5. AsPC-1 — 24h post-PDT
6. AsPC-1+MRC5 — 48h post-PDT (not quantified)
7. AsPC-1 — 48h post-PDT (not quantified)

Phospho-MET (mirrored)

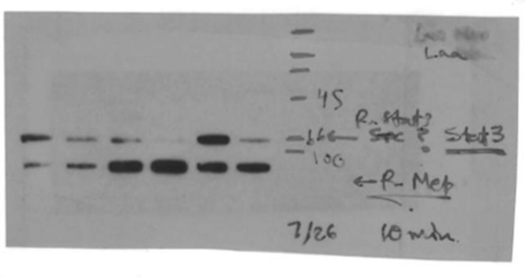

Western Blot – Figure 4
Densitometry

Cell line(s)	Hours post-PDT	Exp. Repeat	β-actin	MET	phospho-MET
**AsPC-1**	0	**1**	15853.51	9067.539	**-**
**AsPC-1**	0.5	**1**	18047.995	5372.104	**-**
**AsPC-1**	4	**1**	19313.53	3375.569	**-**
**AsPC-1**	8	**1**	21504.116	2933.983	**-**
**AsPC-1+MRC5**	0	**1**	17917.388	8730.56	**-**
**AsPC-1+MRC5**	0.5	**1**	16989.51	2972.276	**-**
**AsPC-1+MRC5**	4	**1**	15900.51	2604.69	**-**
**AsPC-1+MRC5**	**8**	**1**	16823.146	3554.347	**-**
**AsPC-1**	**0**	**2**	9979.983	8325.276	4564.033
**AsPC-1**	**1**	**2**	10009.69	3070.962	99.192
**AsPC-1**	**4**	**2**	9426.033	1676.719	401.213
**AsPC-1**	**24**	**2**	8867.497	2026.648	4439.719
**AsPC-1+MRC5**	**0**	**2**	9463.033	8908.74	4254.891
**AsPC-1+MRC5**	**1**	**2**	10140.154	3852.841	1664.87
**AsPC-1+MRC5**	**4**	**2**	9577.033	2440.426	3006.113
**AsPC-1+MRC5**	**24**	**2**	9850.154	3043.305	4878.134
**AsPC-1**	**0**	**3**	**-**	**-**	5593.619
**AsPC-1**	**24**	**3**	**-**	**-**	5859.426
**AsPC-1+MRC5**	**0**	**3**	**-**	**-**	5138.669
**AsPC-1+MRC5**	**24**	**3**	**-**	**-**	8033.033
